# Characteristics and place of death in home care recipients in Germany – an analysis of nationwide health insurance claims data

**DOI:** 10.1186/s12904-022-01060-w

**Published:** 2022-10-06

**Authors:** Rieke Schnakenberg, Alexander Maximilian Fassmer, Katharina Allers, Falk Hoffmann

**Affiliations:** Faculty of Medicine and Health Siences, Department of Health Services Research, Carl von Ossietzky University of Oldenburg, Oldenburg, Germany

**Keywords:** Home care recipients, German health insurance claims-data, Care settings, Place of death

## Abstract

**Background:**

Most care-dependent people live at home, where they also would prefer to die. Unfortunately, this wish is often not fulfilled. This study aims to investigate place of death of home care recipients, taking characteristics and changes in care settings into account.

**Methods:**

We retrospectively analysed a cohort of all home-care receiving people of a German statutory health insurance who were at least 65 years and who deceased between January 2016 and June 2019. Next to the care need, duration of care, age, sex, and disease, care setting at death and place of death were considered. We examined the characteristics by place of care, the proportion of dying in hospital by care setting and characterised the deceased cohort stratified by their actual place of death.

**Results:**

Of 46,207 care-dependent people initially receiving home care, 57.5% died within 3.5 years (n = 26,590; mean age: 86.8; 66.6% female). More than half of those moved to another care setting before death with long-term nursing home care (32.3%) and short-term nursing home care (11.7%) being the most frequent transitions, while 48.1% were still cared for at home. Overall, 36.9% died in hospital and in-hospital deaths were found most often in those still receiving home care (44.7%) as well as care in semi-residential arrangements (43.9%) at the time of death. People who died in hospital were younger (mean age: 85.5 years) and with lower care dependency (low care need: 28.2%) as in all other analysed care settings.

**Conclusion:**

In Germany, changes in care settings before death occur often. The proportion of in-hospital death is particularly high in the home setting and in semi-residential arrangements. These settings should be considered in interventions aiming to decrease the number of unwished care transitions and hospitalisations at the end of life.

## Background

Due to demographic changes the world’s population is ageing and more and more people will die in old age, often affected by multiple chronic diseases and with complex care needs [1]. The official German care statistics from 2019 reported a further significant increase of care dependency to a total of 4.1million people [2, 3]. Four fifths of them were cared for at home. The home care receiving group was younger than the nursing home residents and the proportion of women were with 60% lower than in nursing homes [4]. Accordingly, end-of-life (EOL) care is important in this population and is increasingly being researched. Regardless of the care-setting and the country, the majority of people wish to die at home [5–8], even if one should differentiate between ideal and actual preferred places of care as well as places of death and that both could change over time [9]. A cross-national comparison of places of death including 21 countries of people aged over 65 years from 2013 showed a median of 54% of death in hospital and 18% in residential aged care facilities [10], both with large differences between studies as well as between countries.

Some countries – such as Germany – do not routinely compile data from death registrations including information on care dependency, which makes it difficult to obtain representative data regarding place of death. First data are available for the distribution of places of death in Germany, showing an overall trend in places of death [11]. However, these data do not contain information on care dependency and little is known about care transitions at the EOL in the group of older home care receiving people. Can the people who are cared for at home also die at home, as most would prefer?

Therefore, aim of this explorative study was to investigate place of death of home care recipients in Germany, taking characteristics and changes in care settings into account.

## Method

This retrospective study is part of the STudy on ADvance care PLANning (STADPLAN), funded by the German Federal Ministry of Education and Research (BMBF grant 01GL1707A-D). STADPLAN aims to evaluate the effect of an adapted advance care planning (ACP) program on patients’ activation regarding healthcare issues in care dependent community-dwelling older persons [12].

### Database, study population and outcome

Anonymised data for this study were obtained from the DAK-Gesundheit, a large statutory health and long-term care insurance (LTCI) fund representing approximately 5.6million members (corresponding to 6.7% of the German population) [13]. The dataset included all insured persons being at least 65 years of age and who were cared for in their home setting on January 1, 2016 and contained different datasets that were merged via a unique identifier. For this study, all persons that died up to June 30, 2019 were included. Data on care dependency were obtained from the LTCI [14] providing data on services received with start and end dates and the person’s care need. At baseline all persons included received services in their own home setting (1). According to the received services during follow-up, we differentiate between four further care settings. We included shared housing arrangements (2) where a small group of people is living in private rooms, while sharing a common space, domestic support, and nursing care [15]. Semi-residential arrangements (3) provide a temporary care support during day or night in an institution [16]. Full residential care refers to either short-term stay (4, covered by the LTCI for a maximum of 58 days per year) or long-term stay (5) in a nursing home [17].

Up to 2016, care-dependent persons were assigned to a level of care reflecting the time needed for daily help ranging between 1 and 3. These levels were modified into 5 care grades at January 1, 2017 reflecting a more comprehensive view on the person’s independence and competences considering physical, cognitive or psychological impairments [17]. Different claims data sets also contained information on demographics, date of death, outpatient care, and hospitalisations. Hospital data hold information on dates of admission and discharge, the respective diagnoses and diagnostic as well as therapeutic procedures. Outpatient data contained diagnoses including information on the level of diagnosis certainty (confirmed, suspected, ruled out and status post), treatments and procedures. All diagnoses were coded using the German modification of the international classification of diseases, 10th revision (ICD-10-GM).

Our first outcome of interest was the care setting at time of death differentiated by the above mentioned 5 groups. The second variable of interest was the actual place of death, which in addition to those care settings further includes hospitals. We also assessed the proportion of persons that died in hospital, defined as being in hospital on the day of death. For providing baseline characteristics we assessed the mean age at death (categorised into four groups 65–74, 75–84, 85–94 and ≥ 95 years), sex and care need at death. We combined the old levels and new grades to 3 groups of care need: low (care level: 0/1, grade: 1/2), medium (care level: 2; grade: 3/4), high (care level: 3; grade: 5). Duration of care dependency was calculated as the time in years between the start of receiving care in the home setting (latest January 1, 2016) and the date of death. Furthermore, we assessed confirmed outpatient diagnoses of cancer (ICD-10-GM: C00-D48 [18]) and dementia (ICD-10-GM: F00, F01, F02.0, F02.3, F03, G30, G31.0, G31.1, G31.82, G31.9, R54 [19]) in the quarter of death and the three quarters before death.

### Statistical analysis

Firstly, the study population was described by age, care need, duration of care and care setting at death as well as having a cancer or dementia diagnosis, respectively. These measures were calculated overall and stratified by sex. Secondly, we examined the proportion dying in hospital by their care setting. Finally, we characterised the deceased cohort stratified by their actual place of death (hospital, home setting, shared living arrangement, semi-residential arrangement, short-term care or long-term care in nursing home). Descriptive measures were computed.

We performed all analyses using the SAS programme for Windows, version 9.4 (SAS Institute Inc., Cary, NC, United States).

## Results

The entire cohort encompasses 46,207 care-dependent people, who were cared for at home at January 1, 2016. The target population included 26,590 people, who had died until June 2019 (57.5%).

### Baseline characteristics

Table1 shows the characteristics of all deceased persons plus the comparison between males and females. Two thirds (66.6%) of the deceased cohort were female, more than 80% of all had a medium care need (53.3%) or even a high care need (29.6%) at death. The mean duration of care dependency was 3.7 years (SD: 2.8) at time of death. About two thirds had a dementia diagnosis (64.1%) and one third had a diagnosis for cancer (36.1%). Most of all were cared for at home at time of death (48.1%), followed by the long-term nursing home setting (32.3%) and short-term nursing home care (11.7%).

**Table 1:** Characteristics of deceased cohort (2016 to 2019)

	Total (n = 26,590)	Female (n = 17,701)	Male (n = 8,889)
Age at death in years			
$\stackrel{-}{x}$ (SD)	86.8 (7.4)	87.5 (7.4)	85.2 (7.2)
65–74	1,714 (6.5%)	980 (5.5%)	734 (8.3%)
75–84	7,671 (28.9%)	4,601 (26.0%)	3,070 (34.5%)
85–94	13,517 (50.8%)	9,164 (51.8%)	4,353 (49.0%)
95+	3,688 (13.9%)	2,956 (16.7%)	732 (8.2%)
Care need* at death			
Low (care level: 0/1, grade: 1/2)	4,558 (17.1%)	3,174 (17.9%)	1,384 (15.6%)
Medium (care level: 2; grade: 3/4)	14,173 (53.3%)	9,540 (53.9%)	4,633 (52.1%)
High (care level: 3; grade: 5)	7,859 (29.6%)	4,987 (28.2%)	2,872 (32.3%)
Account for diagnosis assessed in the quarter of death or up to three quarters before death			
Dementia	17,049 (64.1%)	11,461 (64.7%)	5,588 (62.9%)
Cancer	9,603 (36.1%)	5,558 (31.4%)	4,045 (45.5%)
Duration of care dependency at death in years: $\stackrel{-}{x}$ (SD)			
	3.7 (2.8)	3.9 (2.8)	3.4 (2.7)
Setting at death			
Home	12,795 (48.1%)	8,131 (45.9%)	4,664 (52.5%)
Long-term care in nursing home	8,599 (32.3%)	6,109 (34.5%)	2,490 (28.0%)
Short-term care in nursing home	3,103 (11.7%)	2,016 (11.4%)	1,087 (12.2%)
Semi-residential arrangement	1,156 (4.4%)	709 (4.0%)	447 (5.0%)
Shared housing arrangement	937 (3.5%)	736 (4.2%)	201 (2.3%)

* The three German care levels were modified into 5 care grades at 1st January 2017

Females had a higher mean age of 87.5 years at death, compared to 85.2 years at death of all males. Men were more likely to have a cancer diagnosis and a shorter period of care dependency compared to women. Only small differences were found regarding the care needs and the prevalence of dementia. More females received long-term care in nursing homes (34.5% vs. males: 28.0%), whereas more males have been cared for at the home setting (52.5% vs. females: 45.9%).

### In-hospital death

Table2 shows the prevalence of in-hospital deaths in total and stratified by care setting.

**Table 2:** Prevalence of in-hospital death by care setting

	Total (n = 26,590)	Care setting at time of death				
		Home setting (n = 12,795)	Nursing home (long-term care) (n = 8,599)	Nursing home (short-term care) (n = 3,103)	Semi-residential arrangement (n = 1,156)	Shared housing arrangement (n = 937)
Sex						
Male	39.1%	46.3%	28.7%	30.5%	43.2%	34.8%
Female	35.8%	43.8%	26.8%	32.8%	44.3%	23.2%
Age at death in years						
65–74	47.9%	55.2%	29.3%	42.0%	56.4%	45.2%
75–84	42.6%	51.5%	30.9%	35.8%	44.8%	31.2%
85–94	35.2%	42.3%	27.3%	31.1%	44.3%	22.0%
95+	26.2%	31.6%	21.1%	23.1%	29.9%	13.1%
Care need* at death						
Low (level: 0/1, grade: 1/2)	60.7%	71.1%	39.6%	45.4%	75.8%	50.0%
Medium (level: 2; grade: 3/4)	38.4%	47.2%	29.2%	32.5%	53.5%	32.6%
High (level: 3; grade: 5)	20.3%	22.0%	17.5%	20.4%	22.8%	17.1%
Account for diagnosis assessed in the quarter of death or up to three quarters before death						
Dementia	32.6%	39.3%	26.2%	29.8%	40.6%	23.4%
Cancer	36.9%	45.8%	26.2%	29.2%	44.5%	27.1%
Total						
	36.9%	44.7%	27.3%	32.0%	43.9%	25.7%

* The three German care levels were modified into 5 care grades at 1st January 2017

In total, 36.9% of all deceased died in hospital (n = 9,811). The proportion of in-hospital deaths is slightly higher in men (39.1%) than in women (35.8%). With higher age, the proportion of in-hospital deaths decreased (47.9% in the 65–74 years old versus 26.2% in the persons aged 95 or older). Another trend can be seen regarding the care need. More than 6 out of 10 persons with the lowest care need died in hospital compared to one fifth in the group with the highest care need. Differences with respect to dementia and cancer were not found.

The prevalence of in-hospital death also varies by care setting. Whereas less than 28% of deceased cared for in the nursing home (long-term) died in hospital, this proportion was highest in the home care setting as well as the semi-residential arrangement care setting with 44% and 45% each. The sex difference was highest in the shared housing arrangement setting (23.2% in-hospital deaths in the female group versus 34.8% in males). The above-mentioned tendencies regarding age and care needs were found in all care settings. In people with a dementia diagnosis, the proportion of in-hospital deaths varies widely by care setting. It is lowest in the shared housing arrangement setting (23.4%) and the highest in the semi-residential arrangement setting (40.6%).

### Place of death

When having a closer look at the actual place of death (Table3), most persons died in hospital (36.9%), followed by the home setting (26.6%) and the nursing home (long-term) (23.5%). Nearly 13% either died in the short-term care (7.9%), in a shared housing arrangement (2.6%) or in the semi-residential arrangement setting (2.4%).

**Table 3:** Characteristics of deceased cohort by place of death (2016–2019)

	Hospital (n = 9,811; 36.9%)	Home setting (n = 7,077; 26.6%)	Nursing home (long-term care) (n = 6,247; 23.5%)	Nursing home (short-term care) (n = 2,110; 7.9%)	Semi-residential arrangement (n = 649; 2.4%)	Shared housing arrangement (n = 696; 2.6%)
Sex						
Male	35.4%	35.4%	28.4%	35.8%	39.1%	18.8%
Female	64.6%	64.6%	71.6%	64.2%	60.9%	81.2%
Age at death in years						
Mean (SD)	85.5 (7.4)	87.3 (7.7)	88.0 (6.9)	87.2 (7.2)	86.4 (7.1)	86.8 (7.0)
65–74	8.4%	6.4%	4.1%	5.3%	5.2%	4.9%
75–84	33.3%	26.3%	24.8%	26.8%	35.1%	29.7%
85–94	48.5%	51.1%	53.6%	52.6%	47.0%	54.9%
95+	9.8%	16.2%	17.5%	15.3%	12.6%	10.5%
Care need* at death						
Low (level: 0/1, grade: 1/2)	28.2%	11.4%	9.8%	15.6%	4.6%	2.9%
Medium (level: 2; grade: 3/4)	55.5%	45.4%	60.6%	55.4%	41.6%	42.2%
High (level: 3; grade: 5)	16.3%	43.3%	29.7%	29.1%	53.8%	54.9%
Account for diagnosis assessed in the quarter of death or up to three quarters before death						
Dementia	56.6%	60.1%	75.7%	64.6%	81.2%	88.4%
Cancer	36.2%	36.4%	36.1%	40.9%	29.6%	24.7%

* The three German care levels were modified into 5 care grades at 1st January 2017

Overall, 18.8% of those dying in shared housing arrangements were male versus 39.1% in semi-residential arrangements. Deceased people in hospital and semi-residential arrangements were the youngest with 85.5 and 86.4 years in mean, whereas the place of death with the oldest people was the nursing home (long-term) (88.0 years). The prevalence of dementia varies widely between the places of death from highest in shared housing arrangements (88.4%) to lowest at home setting (60.1%) and hospital (56.6%). The highest cancer prevalence was found at the short term-care setting (40.9%) versus lowest in shared housing arrangements (24.7%) as place of death.

## Discussion

### Findings and comparison with the literature

In care-dependent people initially receiving home care, 57.5% died within 3.5 years. Overall, 36.9% died in hospital and in-hospital deaths were found most often in those still receiving home care as well as care in semi-residential arrangements at the time of death. People who died in hospital were younger and had lower care dependency compared to all other analysed care settings. More than half of home care receiving people moved to another (care-) setting before death (44.0% either to long- or short-term care in nursing home, 4.4% to semi-residential arrangements, plus 3.5% to shared housing arrangements).

### Actual place of death

Nearly 37% of all deaths took place in **hospitals**, which is the most common place of death in our care receiving cohort as well as in the total German population [20–22] and in those of most other countries [23–25].

After the **hospital**, the own **home** was found as the second most common place of death with 26.6%, which was also found by Dasch et al. (21.3%) for 2017 although their representative German cohort of all persons dying was in mean 77.6 years old, almost 10 years younger than our care receiving cohort [22]. Moreover, Herbst et al. analysed two random samples of German death certificates from 2007 and from 2017. They showed that while in 2007 home also was the second most frequent place of death (26.1%), it slid to third place in 2017 with 19.8% [20]. Taking results of published studies together, the likelihood to die at home has been decreasing since recent years [20, 22, 26]. In a review of 1998, the authors already investigated the relation between patient characteristics and home deaths [27]. They found out that improved access to home care is likely to increase home deaths for older people [27]. Especially palliative home care and hospice care are associated with fewer hospitalisations and more home deaths [28]. But there are several more potential factors influencing death at home, for example patients functional status, their preferences, living with relatives, and extended family support [29].

The present cohort showed that 23.5% died in nursing homes, which is a little higher than found in the younger-aged representative sample by Dasch et al. (20.4%) but lower than in the random sample of Herbst et al. with 27.1% [20, 30]. The older the people, the higher the probability to die in a nursing home instead of a hospital [31]. Also in our study nursing home residents receiving long-term care were the oldest group.

**Shared housing arrangements** were not considered in previous studies investigating place of death, even if this setting can be seen as an increasingly used, familiar care alternative for long-term nursing homes in Germany [15, 33, 34]. To the author’s knowledge there is also no data on the frequency of transitions to **short-term care** before death as well as nursing home as place of death for short-term care recipients. Studies based on German death certificate data cannot include care information, because they are not routinely covered in these documents. Our study shows that both settings are of relevance and should be included in future studies in EOL care. Quantification and deeper understanding of all possible care transitions at the end of life are important to estimate the relevance and trends for place of death from a public health perspective. Even if the **hospice** as place of death is still rare and unfortunately not covered in the present data, it has been shown to increase as place of death in Germany in recent years [22].

### Hospital death by care setting

As in the present cohort, the proportion of in-hospital death in older, care receiving people seems to be smaller compared to the general population [25]. The availability of formal versus informal care seems to influence hospital death rates. In the Netherlands older people receiving informal care were more likely to die in hospital than people receiving formal home care or institutional care [35]. Another Dutch study showed for Dutch people who only received informal care in their last three months of life that the odds of dying in a hospital was much higher compared to those who received a combination of formal and informal home care [36]. In the present cohort, the proportion of in-hospital death was also highest in people receiving home care (44.7%), where the largest proportion of informal care can certainly be found. The proportion of in-hospital deaths was lower in our group of nursing home residents receiving long-term care. Although this proportion goes in line with previous German analyses [37, 38], it is, however, internationally compared somewhat higher [39]. Nevertheless, the proportion of in-hospital deaths among nursing-home residents internationally varies markedly even within countries with an overall median of 22.6% [39].

There are other possible factors influencing the risk of dying in hospital for elderly, care receiving people like the care level, age and sex. In our cohort, the younger the people and the lower the care level, the higher was the proportion of in-hospital death. The same was found by previous studies [10, 39, 40]. This could possibly partly explain, why men in our cohort were a little more likely to die in hospital than women. However, there is increasing evidence of “real” sex-specific differences in burdensome interventions like transitions of care or invasive procedures during EOL and future studies should put more emphasis on sex-specific analyses [41].

### Moving to other care settings before death

Our result regarding the frequent transition from home to other care settings before death indicates that home care cannot always be maintained until the EOL, although most patients wish so [5–8]. It was already shown in international studies that the frequency of care setting transitions of elderly people increases near to death [42]. For example, in the Netherlands nearly half of their 55–85 years old home-living persons was transferred between care settings one or more times in the last 3 months of life, mostly from home to hospital [35]. Care setting transitions at the EOL are seen as increasingly problematic, also because of potential medication and care errors, disrupting care teams, and a loss of care information [20, 27, 31], even if these transitions have the potential to be a relief for family caregivers [23]. Looking at the German situation, it also should be mentioned that structures in outpatient palliative care have been introduced just within the last 15 years and the growing number of general and specialist outpatient palliative care services (in German: AAPV and SAPV) provides more possibilities of outpatient palliative care since the last years [43], which can strengthen the quality of care at home at the patient’s EOL. Therewith unwanted care transitions can also be prevented. However, the care at home should not automatically be equated with the best care [9, 44] because institutionalised palliative care like hospice care and in-hospital palliative care can improve the quality of dying and death [45, 46]. Overall, care decisions always should be weighed individually to enable appropriate and timely care setting transitions in accordance with individualised EOL care needs [47].

There are different indicators already mentioned being associated with a risk of care transitions. In the UK, that people with severe cognitive impairment were the most likely group to move to other care settings [42]. Our results also show that the people with dementia more often died in another care setting than home. Another German study analysed predictors of admission to nursing home in care dependent people based on longitudinal secondary data and also found dementia, cognitive impairment, cancer of the brain and higher age as risk factors, which goes in line with our results [48].

### Strengths and Limitations

The strengths of this study are its real-world character, its large sample size which allowed us to stratify the analyses by sex, age and other variables. Furthermore, we had valid information on care setting pathways and place of death. Just like the strengths, the limitations are based to the nature of the administrative data from LTCI funds. The data were not captured for the purpose of scientific research and further information that could influence the placement and dying in different care settings (clinical data, socioeconomic status, marital status or family support, respectively) were not available. The same applies to further information related to the specific care recipient’s institution, e.g. staffing ratios or the nursing home’s ownership. For the ones living in semi-residential arrangements we were not able to differentiate between the care-recipients died at home or during day or night care, respectively. For the shared housing-arrangements an underreporting is possible since in this case care providers might only invoice other benefits for instance for nursing home care. Besides, our data did not contain palliative care units and hospices as places of death. However, their joint proportion on places of death in Germany is 11% [22]. Another limitation relates to the fact that data for this study were only obtained from one health insurance fund. Since the DAK-Gesundheit insures more women and a population with a generally poorer health status [49], our results cannot be extrapolated to the entire care receiving population in Germany. Nevertheless, the DAK-Gesundheit is with 5.6million insured persons one of Germany’s largest health insurance funds [13].

## Conclusion

In a large cohort of persons that were initially cared for at home, more than half moved to another care setting before death, most often to long-term nursing homes. Overall, about 4 of 10 perons died in hospital with highest proportions in those still receiving home care as well as care in semi-residential arrangements. Thus, there are still many unwanted and potentially preventable care transitions at the EOL in Germany. Interventions are needed to improve EOL care both in professional as well as informal home care settings also including semi residential arrangements. For example, ACP interventions were already proved effective as well as to support informal caregivers. Moreover, outpatient palliative care should be improved. This means, inter alia, an extension of an ACP-offer to the home setting in Germany as well as better access to outpatient hospice and palliative care services.

## Data Availability

The data that support the findings of this study are available from the DAK-Gesundheit but restrictions apply to the availability of these data, which were used under license for the current study and are not publicly available. There are no linked research data sets for this paper. The authors do not have the permission to share data.

## Abbreviations

ACP: Advance care planning.
EOL: End of life.
ICD-10-GM: German modification of the international classification of diseases, 10th revision.
LTCI: Long-term care insurance.
STADPLAN: Study on advance care planning.
